# Book Review: “Classifying Reality,” by David S. Oderberg (ed.) (2013)

**DOI:** 10.3389/fpsyg.2015.00461

**Published:** 2015-04-16

**Authors:** Paul M. W. Hackett

**Affiliations:** School of Communication, Emerson CollegeBoston, MA, USA

**Keywords:** classification, categories, mapping sentence, facet theory, mereology, structural ontology, psychological processes, book reviews as topic

Classifying reality is an ancient and fundamental ability. Even the most basic living creatures classify their world as do social collectives of living things, most noticeably, human beings through psychological processes. An example of the rudimentary nature of classification exists in the behavior of slime molds. When food is abundant many of the more than 900 types of slime molds exist not as slime but as single-cell organisms. However, if food is in short supply the cells agglomerate and move as a single body. As a collective, slime molds are able to identify food and experiments have demonstrated that through changing shape the mold can reach food in a maze: Branches of the mold that do not terminate at a food source “die off” resulting in an almost singular path to food. Remarkably, these single cell organisms can act as a collective and classify correct or incorrect, advantageous or non-advantageous turns in the maze. At this rudimentary behavioral level slime mold is able to classify reality in order to bring advantage to the collective. This example demonstrates the fundamental nature of classification as a behavioral and biological process. Within psychology classifying has featured large within many areas of the literature but perhaps mainly within developmental and cognitive sub-disciplines.

Notwithstanding the seminal nature of the process of classifying in human life, contemporary books published on the subject of classification are uncommon and I believe that psychologists could benefit from considering the broad perspective assumed in Oderberg's book. His edited volume is a concise collection of writing by contemporary scholars in which each of the six chapters is concerned with an aspect of identifying the structure of reality. Realism proposes that some entities do not need conceptual systems, beliefs, or our linguistic practices, etc., and may be thought of as objectively real. This contrasts with conventionalism, which posits entities are constituted on social agreement. The book contains an eclectic assemblage of writing from authors drawn from a realist perspective as, “… realism about classification stands its ground: all major lines of criticism available to the extreme conventionalist can be addressed.” Tahko (2013, p. 60).

The central question posed in Oderberg's book may be summarized as: Is it possible to classify reality? Here he makes the not universally accepted point that in order to be able to classify reality it is necessary that we establish that reality has clearly extant boundaries to its content. Oderberg's collection does not specifically concentrate upon categories per-se, but rather upon classification, where amongst other questions he asks of the extent to which classification is a fabrication of the mind? However, due to limited space and the title of this special edition I will emphasize categorization. Essays in the first section of the book each chapter reviews somewhat abstract questions and issues associated with classification within a realist approach. The authors question any general or universal framework for classifying being or the development of a general ontology. Oderberg presents theoretical notions of classification as presented in the late E. J. Lowe's opening chapter on categorical predication. This chapter offers a synopsis and extension of Lowe's thinking on his four-category ontology (Lowe, 2006). Lowe asks how many components would be needed to adequately define a basic categorization of our world and proposes this to be four where any elements within such a system must be mutually exclusive in what they define and that their content cannot be classified through the combination of other elements in the ontology. In a revision of formal logic, Lowe presents a complex categorical ontology of the kinds of things that form the fundamental components of our world. Tahko, in his chapter, questions the conventional notion that there is no best or more realistic form of classification of the fundamental nature of the world. In section two the authors put forth their thoughts on the use of objective classification in science in general and specifically in biology. Authors develop a more applied understanding of classification in chapters such as Stephen Boulter's that posit thoughts upon the classification of biological forms and kinds and in Rosenkrantz's writing that forwards the notion that there are necessary and essential aspects of living things.

In the above review I have demonstrated the breadth and depth of the chapters' contents and I will now evaluate Oderberg's text as this may be applicable to psychologists. Whilst the book offers a diverse range of thinking on the process of classification there is no concluding chapter on contemporary thinking on classification and its further development and usage. Furthermore, the authors pay little heed to the relationship between classification units (mereology—the study of part-to-part and part-to-whole relationships) either as independent component of classification or as componential sub-units. My work in this area using the mapping sentence as a mereological framework (see, Hackett, 2014) is pertinent in this regard where I suggest that not all classification forms (or categories) are, or should be thought of, as equal. The mapping sentence I put forth as a flexible framework for the incorporation of the combined effects of inter-related or non-independent categories or classifications in a meaningful manner.

It is appropriate to review this book in this specials edition on the psychological aspects of categorization as psychological approaches associated with categorization may be identified as a special case of classification. Indeed, the title of this book *Classifying Reality* could have appropriately been the title of this special edition. The psychology study of classification and categories as a separate area of research is neglected and lacks a clear theoretical conception of categorizing processes. Oderberg's volume offers psychologists, and others, an insightful starting point from which to develop research into classification especially when this is read and employed in conjunction with the psychological approach of the mapping sentence.

## Conflict of interest statement

The author declares that the research was conducted in the absence of any commercial or financial relationships that could be construed as a potential conflict of interest.
